# Estimating resource selection with count data

**DOI:** 10.1002/ece3.617

**Published:** 2013-06-07

**Authors:** Ryan M Nielson, Hall Sawyer

**Affiliations:** Western EcoSystems Technology, Inc.Laramie, Wyoming, 82070

**Keywords:** Generalized linear model, habitat use, overdispersion, panel data, Poisson regression, resource selection probability function

## Abstract

Resource selection functions (RSFs) are typically estimated by comparing covariates at a discrete set of “used” locations to those from an “available” set of locations. This RSF approach treats the response as binary and does not account for intensity of use among habitat units where locations were recorded. Advances in global positioning system (GPS) technology allow animal location data to be collected at fine spatiotemporal scales and have increased the size and correlation of data used in RSF analyses. We suggest that a more contemporary approach to analyzing such data is to model intensity of use, which can be estimated for one or more animals by relating the relative frequency of locations in a set of sampling units to the habitat characteristics of those units with count-based regression and, in particular, negative binomial (NB) regression. We demonstrate this NB RSF approach with location data collected from 10 GPS-collared Rocky Mountain elk (*Cervus elaphus*) in the Starkey Experimental Forest and Range enclosure. We discuss modeling assumptions and show how RSF estimation with NB regression can easily accommodate contemporary research needs, including: analysis of large GPS data sets, computational ease, accounting for among-animal variation, and interpretation of model covariates. We recommend the NB approach because of its conceptual and computational simplicity, and the fact that estimates of intensity of use are unbiased in the face of temporally correlated animal location data.

## Introduction

Resource selection, or use, is typically determined by comparing characteristics of used locations to available ones (Manly et al. 2002) using weighted distribution theory (Johnson et al. 2006) and an exponential resource selection function (RSF; Manly et al. 2002). Other methods for comparing used to available locations include distance-based analyses like Mahalanobis (Clark et al. 1993) and maximum entropy density estimation (Royle et al. 2012). All these approaches treat use as a binary response (1 = used; 0 = available), even when it is not known whether available locations were actually unused (Keating and Cherry 2004; Johnson et al. 2006). When animal locations are collected at fine spatiotemporal scales using global positioning systems (GPS), we suggest an alternative approach that analyses intensity of use. Counts of use across the landscape can be modeled using negative binomial (NB; Hilbe 2008) regression (e.g., Sawyer et al. 2006, 2009). Given a sufficient and representative sample of animal locations, the frequency of use in a sample of “habitat” units can be regressed against the characteristics (covariate values) of those units. Thus, rather than treating use the same, regardless if the site was used once or multiple times, this NB approach quantifies use along a continuum that ranges from zero to the maximum number of observed animal locations in a sampling unit.

Other advantages of the NB approach include: (1) the flexibility to estimate RSFs that represent either relative (RSF) or true (RSPF) probability of use, (2) the ability to produce estimates of resource selection that are unbiased in the face of serial correlation in the location data, and importantly, (3) it is easily implemented with basic statistical packages and is not computationally intensive.

Here, we describe a common sampling situation where animal locations are recorded via GPS collars, and show how the intensity of use for a set of sampling units can be related to the habitat characteristics of those units with NB regression. We demonstrate use of NB regression with GPS location data from 10 elk. In addition, we discuss model assumptions, interpretation of model coefficients, and illustrate how to account for among-animal variability in estimates of precision.

## Methods

### Sampling

**Figure 1:**
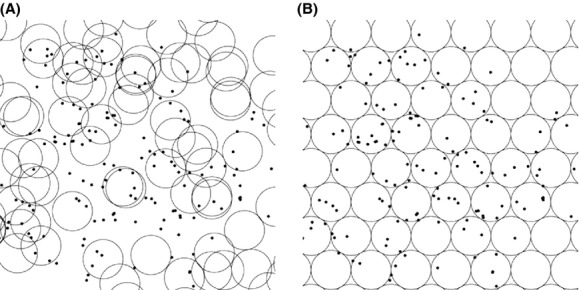
A random sample with replacement of circular sampling units (A), and a systematic sample of circular sampling units based on a random start (B), with hypothetical animal locations.

Consider a sampling situation where a GPS collar collects an animal's locations at a fixed sampling intensity (e.g., one location every 3 h) to provide *T* locations over a specific period of time. Also suppose that the two-dimensional space available to the collared animal is correctly identified, and a random sample of *i* (*i* = 1, 2, …, *n*) sampling units is drawn from the available space. The sampling units should have complete spatial coverage across the study area, but can be of any shape (e.g., rectangular, circular, hexagonal), provided they are all the same size (e.g., circular units with 200-m radii). A simple random sample with replacement of sampling units (Fig. 1) is a common method for spatial sampling, but can result in clumps of overlapping units or gaps between sampled areas. Sampling units that are systematically spaced with a random start (Fig. 1) will generally provide a better representation of the study area and in some circumstances will improve precision (Manly 2009).

Regardless of the shape of the sampling units and whether they are selected by a random or systematic sample, it is important to note that the total count of animal locations across the sampled units should not be guaranteed to sum to *T*. If the total count of animal locations within the selected sampling units is known, this represents multinomial sampling rather than Poisson sampling (Ramsey and Schafer 2012). Multinomial sampling requires the more cumbersome multinomial model that uses multiple equations to estimate the probability of each outcome – that is, 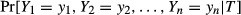 (Cameron and Trivedi 1998). As the multinomial model uses multiple equations it requires larger sample sizes, and interpretation can be difficult. Thus, for the purposes of RSF estimation we recommend a Poisson sampling approach where the total sum of counts within the sampling units is not known in advance, and a Poisson or NB distribution can be used for modeling. A simple random sample of potentially overlapping sampling units, or a systematic sample with a random start of nonoverlapping sampling units with unsampled areas in between will ensure that the counts of animal locations within all the sampling units is not guaranteed to sum to *T* (Fig. 1). Furthermore, locations included in more than one sampling unit or locations not captured in any of the sampling units will not bias model coefficients, as they are randomly determined under the proposed method.

Evaluation of many factors can help determine an appropriate size for the sampling units, but in general, the sampling units should be small enough to detect changes in animal movements while providing counts of animal locations that approximate a NB distribution (see Assumptions). Most importantly, the error in the animal locations and the derived covariates should be considered when determining an appropriate sampling unit size. Sampling units should be larger than the expected spatial error in the animal locations and covariate values. We recommend investigating multiple sizes of sampling units prior to modeling, although we caution against data mining for statistically significant results.

### Statistical model

The number of animal locations (not the number of animals) in each sampling unit provides an unbiased measure of the frequency of animal use during the study period, provided environmental conditions such as canopy cover or terrain ruggedness do not prevent *T* locations from being recorded (Nielson et al. 2009). Count data from Poisson sampling are typically modeled using Poisson or NB regression (Cameron and Trivedi 1998). Poisson regression presumes a vector *Y* of non-negative counts with a variance that is equal to the mean (i.e., E[*Y*] = *u*, and var(*Y*) = *u*). However, because observed count data almost always have a variance larger than the mean (White and Bennetts 1996; Cameron and Trivedi 1998), NB regression usually provides a better representation of observed count data because it allows the variance to exceed the mean. Various NB model parameterizations exist, and distinctions are made based on the link function used and the assumed distribution of var(*Y*). For example, a NB1 formulation (log link) assumes var[*Y*] = *u* + *u*/*θ*. Theta (*θ*) is often referred to as the dispersion parameter, but in the NB1 formulation *u*/*θ* is the amount of overdispersion, or additional variation in the data relative to a Poisson distribution. One might typically assume *θ* is constant, but it could also be a function of the habitat characteristics and modeled as such, although supplying a covariate model for *θ* is not a standard option in many NB regression model fitting routines and we do not discuss it further here.

The NB2 (log link) is the most common parameterization (Cameron and Trivedi 1998), which specifies that var[*Y*] = *u* + *u*^2^/*θ* (Hilbe 2008). The NB2 regression model is represented by


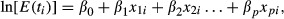
(1)

where *t*_*i*_ is the total number of GPS locations within sampling unit *i* during the study period, β_0_ is an intercept term, β_1_, β_2_, …, β_*p*_ are coefficients to be estimated, *x*_1*i*_, …, *x*_*pi*_ are the values of *p* covariates measured on sampling unit *i*, and *E*[.] denotes the expected value.

Modeling counts of use is acceptable, but it is often preferable to make inference to the relative frequency distribution of animal locations within the study area during the study period, also known as the utilization distribution (UD; Kernohan et al. 2001). With a slight adjustment to the NB model (eq. (1)) we can change the response from counts to relative frequencies. Inclusion of an offset term, ln(*T*), in equation (1) results in


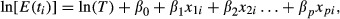
(2)

which is equivalent to


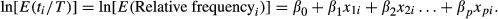
(3)

Thus, the offset term simply scales the response to ensure modeling the relative frequency of use (e.g., 0, 0.003, 0.006, …) instead of integer counts (e.g., 0, 1, 2, …). Because the standard definition of probability is “the long run relative frequency of occurrence,” we can also refer to predictions of relative frequencies as estimates of probabilities of use by the monitored animal(s) during the study period.

Predictions using equation (1) represent counts of use. Predictions from equation (3) represent relative frequencies, or approximations of probabilities of use and thus the model is considered a resource selection probability function (RSPF; Manly et al. 2002). If the sampling units are allowed to overlap or spatial gaps exist between the units (Fig. 1), predictions are not subject to a unit-sum constraint, but should be within the (0, 1) interval.

Standard statistical software packages such as R (R Development Core Team 2012) and SAS (SAS Institute, Cary, NC) can easily fit NB regression models. These software packages estimate NB regression models using maximum likelihood (ML), or a simplification allowed for exponential-based models using iteratively reweighted least-squares algorithms. Thus, information theoretic approaches such as Akaike's Information Criteria (AIC; Burnham and Anderson 2002) can be used for variable selection or to compare competing models.

### Assumptions

A key assumption to any study of resource selection is that the sample of animal locations is representative of the group of animals for which inference is desired. For GPS studies where fix-rate success is poor and the realized number of recorded locations is <*T* (i.e., Pr[detection] <1), then alternative approaches may be needed to account for missing locations and habitat-induced fix-rate bias (Frair et al. 2004; Nielson et al. 2009; Augustine et al. 2011). Another obvious, but easily overlooked assumption of the proposed modeling approach is that the counts from the sampling units follow the NB distribution chosen (e.g., the NB2 distribution). We discuss three assumptions for application of most NB distributions, although some assumptions can be relaxed for certain distributions (e.g., truncated NB, zero-inflated NB). First, the data cannot be censored or truncated. Violation of this assumption is not anticipated in the RSF setting, as it would only occur when a portion of the locations are ignored (e.g., counts >7 are set to 7). However, truncated NB models are available if necessary. For example, if the sampling design does not permit the possibility of zero counts, a zero-truncated NB model (Hilbe 2008) should be used. Second, the sample units should not contain an excess of zeros. Unfortunately, there is no clear rule for how large the counts can be or what constitutes an excess of zeros. Most NB distributions can allow for very large counts and many zeros. For example, based on a NB2 distribution with a mean of *u* = 5 and *θ* = 0.2, the probability of getting a zero count within a sampling unit is 0.52, and the probability of getting a count >50 is 0.013.

Alternative models have been developed to accommodate an excessive number of zero counts, including the hurdle and zero-inflated NB models (Hilbe 2008). Both of these models assume that the data can be separated into two distributions. The hurdle model assumes that a binary process determines whether a count should be >0, and then a count process generates the actual count (≥1) for those units. The zero-inflated NB model allows for modeling of zero counts using a mixture of binary and count processes – that is, zeros could have come from either the binary or count process. Use of either the hurdle model or the zero-inflated NB requires assuming that some of the responses have to be zero, regardless of sampling intensity (fix schedule and/or study length). This implies that either all (hurdle) or a portion (zero inflated) of the sampling units with zero counts were either not available to the study animal(s), or that it was not possible to detect the animal(s) in those sampling units. Obviously, use of these models requires thoughtful justification which should not be based solely on the proportion of zero counts in the data. Proper identification of what is available to the animal may preclude use of these more complicated models.

The third assumption deals with independence and requires that data are not structured as panels (clusters; Hilbe 2008). For example, longitudinal data (GPS fixes) often come in panels (Hilbe 2008), and each sampled animal represents a panel, provided the sample of animals was not clustered (e.g., several animals from same group). In this situation, we may expect spatial correlation in habitat use within each panel, and “within-panel correlation will result in overdispersed data,” (Hilbe 2008) which will result in underestimation of model SEs and inflated Type I error rates. An informal goodness of fit can reveal the potential for panel data. This test involves comparing the residual deviance, *D*, to the model degrees of freedom (df = *n* − [*p* + 1]), with *D* significantly greater than df being evidence of groupings in the data or other potential violations of the other assumptions listed above. Accounting for panel data in this sampling scheme is the same as treating the animal (or family group) as the “experimental unit” (Thomas and Taylor 2006). This can be done by fitting a separate model to each experimental unit and then averaging coefficients and calculating the SE of the mean coefficient (e.g., Marzluff et al. 2004; Sawyer et al. 2006). If some animals, by chance, have too few locations within the selected sampling units to fit equation (3) (e.g., the counts do not follow a NB2 distribution), we recommend use of other methods such as random-effects models, generalized estimating equations, and bootstrapping (see Discussion).

Finally, we emphasize that temporal independence of the animal locations is not a requirement of the NB RS(P)F presented here because the sampling units provide the response variable, and those units have no associated time stamp other than the period in which the study was conducted. Computationally, this is an appealing attribute of the NB RS(P)F approach because temporal correlation is not considered a nuisance, nor does it have to be explicitly modeled (see Fieberg et al. 2010). We note, however, that animal locations should be sampled at the same temporal frequency throughout the period of interest (i.e., similar fix-rate schedule), unless the locations are weighted appropriately (Fieberg et al. 2010).

### Example

We illustrate the NB RSPF using 4,911 locations from 10 GPS-collared Rocky Mountain elk (*Cervus elaphus*) in the Starkey Experimental Forest and Range (hereafter Starkey), the site of long-term ungulate research within a landscape-scale enclosure of 7768 ha (Wisdom et al. 2005) in northeastern Oregon, USA. This research was conducted following review and approval by the Starkey Institutional Animal Care and Use Committee, as required by the Animal Welfare Act of 1985 and its regulations. Researchers specifically followed protocols established by the Starkey Institutional Animal Care and Use Committee for conducting deer and elk research at Starkey (Wisdom et al. 1993).

The store-on-board GPS collars (GPS 4400M; Lotek Wireless Inc., Newmarket, Ontario, Canada) were programed to attempt location fixes every hour between 1 August and 17 August 2010, and then every 5 min from 18 August through 21 August 2010. For this example, we used all location data prior to 18 August along with the first location obtained every hour after 17 August, resulting in a sample of locations with consistent coverage during the study period. The GPS fix success was >99%, and 94% of the locations were three-dimensional.

To estimate frequency of use we took a systematic sample of 502 nonoverlapping circular sampling units with 200-m radii and calculated the number of elk locations within each unit. Because the sampling units were much larger than the expected error in the GPS locations (<20 m), we were not concerned about the location error affecting model results. For this example, we fit a regression model with four covariates: (1) distance to nearest road, (2) mean percent slope, (3) distance to cover-forage edge, (4) and mean soil depth (cm). All distances were measured in km from the center of each circular sampling unit and based on landscape features within 4 km of the study area. Thus, we considered that roads and cover-forage edges directly outside of the enclosure could have affected the distribution of elk.

We estimated a NB RSPF using the glm.nb function based on the NB2 formulation available in the MASS contributed package (Venables and Ripley 2002) for the R software. The offset term in the model was ln(4911), which represents the natural log of the total number of recorded locations summed over all animals. We chose to pool the data across animals to estimate model coefficients and account for among-animal variation by bootstrapping the individual animals 1000 times, making the RSPF a marginal model where the variability between and within animals is collapsed. The SD of the 1000 estimates for each parameter was our estimate of the SE for each coefficient, and the central 90% of the distribution for each coefficient was used as the 90% CI (Manly 2007).

Of the 4911 elk locations collected, 4240 occurred within the 502 sampling units. The mean count of locations in the sampling units was 8, and ranged from 0 to 105. Approximately 31% of the sample units had 0 animal locations. Based on the model fit to the count data, the ML estimate of *θ* = 0.424. A NB2 distribution with these parameters should have, on average, 27% zero values, which is close to the observed proportion. The ratio of the observed residual deviance to the residual df was 1.1, indicating minor overdispersion not explained by the model, which we expected due to the panel nature of the location data.

Estimates of coefficients (Table 1) for the regression model suggest that intensity of use was highest in areas with steeper slopes, deep soils, away from roads, and close to the cover-forage edge (Table 1). Standard errors based on bootstrapping were generally more conservative (larger) than those estimated using the standard model output ignoring the panel nature of the counts (Table 1).

**Table 1:** Elk resource use modeling results, with coefficients, SEs based on maximum likelihood (ML) and bootstrapping, and 90% percentile confidence intervals based on bootstrapping

Covariate	Estimate	ML (SE)	Bootstrap (SE)	90% Confidence interval	

				Lower limit	Upper limit
Intercept	−8.025	NA	NA	NA	NA
Distance (km) to road	0.545	0.172	0.277	0.141	1.041
Mean % slope	0.002	0.008	0.018	−0.026	0.033
Distance (km) to cover-forage edge	−0.355	0.284	0.369	−0.985	0.246
Mean soil depth (cm)	0.022	0.003	0.003	0.018	0.028

**Figure 2:**
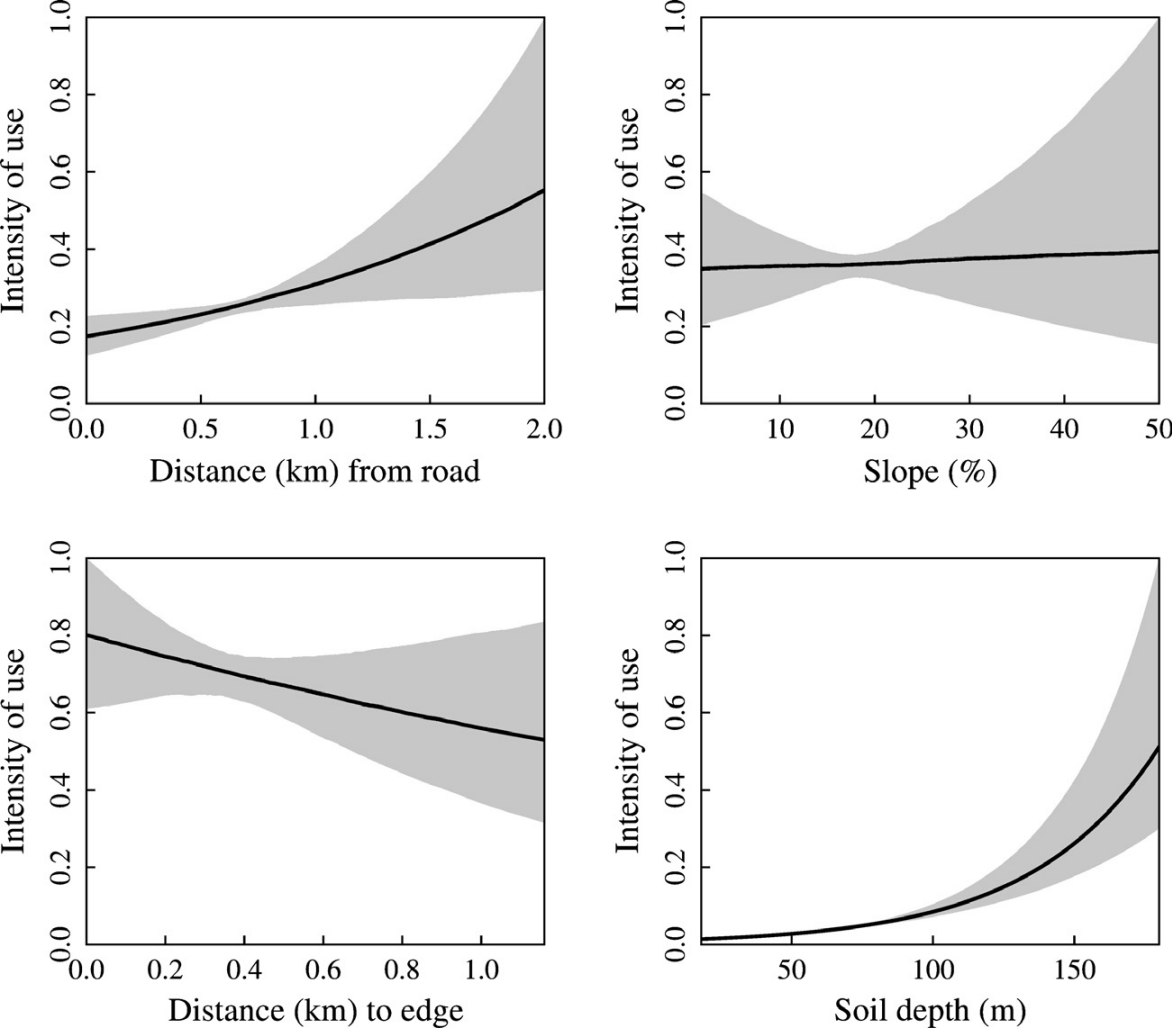
Predicted intensity of use by elk as a function of covariates in the negative binomial RSPF based on 1000 bootstrap replicates treating the individual animal as the experimental unit. Solid lines represent the median predicted value for each level of the covariate. Shaded areas represent 90% confidence intervals (CIs) based on the 0.05 and 0.95 percentiles of the 1000 bootstrap replicates. Median and 90% CI predictive intervals in each panel were scaled to have an overall maximum value of 1.0. Levels of covariates not plotted were held constant at their median values.

The odds ratio for distance from road indicated that probability of elk use was expected to increase by 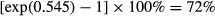 for every 1 km increase in distance from road out to a maximum of 2 km. The odds ratio for mean slope indicated that for every 1-unit increase in mean % slope between 0 and 50% there was an expected 0.24% increase in elk use. Odds ratios also indicated that probability of elk use was expected to decline by 30% for every 1 km increase in distance to cover-forage edge, out to a maximum distance of 1.2 km, and elk use increased by 2.23% for every 1 cm increase in soil depth between 0 and 185 cm. Marginal plots (Fig. 2) illustrate how predicted elk use changed across the range of the observed data. Ninety percent prediction intervals were calculated using the 1000 bootstrap replicates to create a distribution of predictions for each level of each covariate.

## Discussion

Advances in GPS telemetry allow researchers to collect enormous amounts of location data from individual animals at fine spatiotemporal scales. We suggest that analyzing these types of data with the NB RSPF may be preferable to traditional approaches (e.g., logistic regression) that model use as a binary response, unless only a small number of animal locations are recorded and the counts of use within sampled units do not adhere to a NB distribution.

First and foremost, the NB RSPF takes advantage of frequent animal locations by modeling the relative frequency of animal locations across the sampled units. Frequency of use in this scenario is defined by the total number of animal locations within an area during the study period, but does not distinguish between an animal remaining at one location for a long time versus an animal returning repeatedly to the same location.

This simple approach to modeling relative frequency of use is a surrogate for estimating and modeling a UD surface. This approach is similar to the resource utilization function (RUF; Marzluff et al. 2004), and has been shown to provide complementary results, although a Poisson rather than a NB RSPF was used in that evaluation (Millspaugh et al. 2006). However, the NB approach has a few advantages over the RUF in that it allows for nonconstant error variance and does not require first estimating a UD using a kernel density estimator (KDE) prior to modeling a RSPF using habitat covariates, which simplifies estimating variation in the model coefficients (Millspaugh et al. 2006).

When multiple animals are sampled from a population it is necessary to account for among-animal variation in order to make population-level inference (Thomas and Taylor 2006; Fieberg et al. 2010). This can easily be done in the NB RS(P)F framework by using a two-stage approach (Fieberg et al. 2010), where a separate model is fit to each animal (stage 1), and then coefficients are averaged across animals and SEs for the population-level model are estimated using the sample of coefficients (stage 2; e.g., Marzluff et al. 2004; Sawyer et al. 2006). Two-stage approaches that involve averaging coefficients from a NB or other generalized linear model produce an RSF (as opposed to RSPF) and predictions that are equivalent to a geometric average of the predictions of the individual models. Three potential disadvantages with the two-stage approach include: (1) because each model must have the same covariates, there is no standard method for model selection (i.e., AIC), (2) the coefficients of each animal contribute equally to the population-level model (Thomas and Taylor 2006) regardless of the number of locations provided by each animal, unless individual weights are incorporated at the second stage, and (3) some animals, by chance, may have too few locations within the selected sampling units to fit equation (3) to each individual (e.g., the counts do not follow a NB2 distribution).

Perhaps a better method is to pool the data across animals and treat the animal as the experimental unit by bootstrapping (Manly 2007) the individual animals (e.g., McDonald et al. 2006; Goldstein et al. 2010). When using NB regression, this method has several advantages over the traditional two-stage approach, including: (1) the final model is a RSPF rather than RSF, (2) the contribution of each animal is automatically weighted by the number of locations, and (3) various models can be fit and evaluated with standard IT approaches to model selection. Alternatively, we recognize that the sampled animals could be treated as random effects in a mixed-effects model using either frequentist (Gillies et al. 2006; Aarts et al. 2008) or Bayesian (Thomas et al. 2006) methods, or robust sandwich estimators with generalized estimating equations (GEE; Fieberg et al. 2009; Koper and Manseau 2009). However, these methods are inherently more complicated and rely on proper specification of correlation structures (Fieberg et al. 2010), and large numbers of sampling units and/or marked animals may preclude these approaches due to excessive computation requirements.

Regardless of whether the NB is used to estimate individual- or population-level models, the coefficients are easily interpreted through odds ratios and marginal plots. In addition, it is often helpful to map model predictions to visualize changes in habitat use and identify key habitats within the same or similar study area at different points in time (Johnson et al. 2004; Fortin et al. 2005; Sawyer et al. 2009). When mapping predictions for a new set of sampling units, it is important to realize the sum of NB RSPF predictions that will not necessarily sum to 1, and thus rescaling may be necessary if a map of probability of use is desired. In order to improve the resolution of the map, it is valid to make predictive maps at a finer scale compared with the original sample of units used to estimate the RSPF. However, the size and shape of the sampling units should be consistent from modeling to mapping. For example, Sawyer et al. (2009) used 4500 randomly selected circular sampling units with 100-m radii to estimate resource use by mule deer. Their predictive map was then generated on a 104 × 104 m grid covering the study area. Covariates in the final RSPF were measured on 100-m radii circular sampling units centered on each cell in the 104 × 104 m grid. Thus, their circular sampling units used for the predictive map overlapped. Regardless of how a predictive map is created or for what spatial extent, it is important to remember that predictions should be within the range of the modeling data (Neter et al. 1996).

Contemporary analysis of resource use often involves large data sets collected at fine spatiotemporal scales. The NB RS(P)F method provides an attractive framework to analyze such data because animal use is modeled along a continuum, temporally correlated location data do not bias estimates, and among-animal variability is easily accounted for such that marked animals are correctly treated as the experimental unit. Additionally, the conceptual and computational simplicity of the NB RS(P)F makes this approach appealing for a wide range of practitioners from graduate students to experienced statisticians.
